# Human Kallikrein 2: A Novel Lineage-Specific Surface Target in Prostate Cancer

**DOI:** 10.1158/1078-0432.CCR-25-0950

**Published:** 2025-07-08

**Authors:** Fei Shen, Ryan Smith, Theresa McDevitt, Krista Menard, Shaozhou Tian, Gerald Chu, Ruchi Chaudhary, Jennifer McCann, Halley Oyer, Sherry C. Wang, Steven Max, Peter Francis, William K. Kelly, Charles G. Drake

**Affiliations:** 1 Johnson & Johnson, Spring House, Pennsylvania.; 2 Johnson & Johnson, San Francisco, California.; 3 Johnson & Johnson, San Diego, California.; 4 Johnson & Johnson, Raritan, New Jersey.; 5 Thomas Jefferson University, Sidney Kimmel Comprehensive Cancer Center, Philadelphia, Pennsylvania.

## Abstract

**Purpose:**

Targeted therapies for metastatic prostate cancer are limited, highlighting the need for novel drug targets and mechanisms of action (MoA). Human kallikrein 2 (KLK2) is a prostate-specific antigen expressed across the prostate cancer disease continuum. However, it was not recognized as a therapeutic target for prostate cancer in the past due to limited evidence of its cell surface expression. In this study, we systematically characterized KLK2 expression in prostate cancer, confirmed its cell surface expression, and demonstrated the preclinical efficacy of three KLK2-targeting therapeutics with distinct MoA.

**Experimental Design:**

The KLK2 expression profile in different stages of prostate cancer and its cell surface expression were confirmed by IHC and multiplex immunofluorescent staining. The preclinical efficacy of three KLK2-targeting therapeutics was characterized using *in vitro* prostate cancer cell lines, patient-derived material, and *in vivo* xenograft mouse models.

**Results:**

KLK2 was found to be robustly and homogeneously expressed in localized prostate cancer and metastatic hormone-sensitive prostate cancer, whereas some heterogeneity was observed in the visceral lesions of metastatic castration-resistant prostate cancer. KLK2 expression was more specific than that of other prostate cancer target antigens. Although KLK2 is traditionally described as a secreted protease, our results demonstrated its cell surface expression in both prostate cancer cell lines and patient-derived tumors. Notably, targeting KLK2 with three different MoAs, including bispecific T-cell redirector, targeted α-radioligand, and autologous chimeric antigen receptor T cells, showed potent *in vitro* activity and robust *in vivo* tumor control.

**Conclusions:**

Our study establishes KLK2 as a highly prostate-specific cell surface target. Targeting KLK2 with various MoAs represents novel therapeutic approaches for advanced prostate cancer.

*See related commentary by Blinka and Yu, p. 4393*

**Translational Relevance**

Prostate cancer is the fourth most common cancer in men. Metastatic prostate cancer is incurable, and only about a third of patients with metastatic disease survive longer than 5 years. Effective, life-prolonging targeted treatments for advanced prostate cancer remain an urgent unmet need.

Human kallikrein 2 (KLK2) is expressed exclusively in the prostate, and our data show that high and specific expression is maintained at different stages of prostate cancer. More importantly, we also confirmed that KLK2 is localized on the cell surface, making it an attractive surface target in prostate cancer. Our *in vitro* and *in vivo* experiments suggest that KLK2 can be effectively targeted by a bispecific T-cell engager, a radioconjugate antibody, and chimeric antigen receptor T cells, with robust antitumor activities in mouse xenograft models. These findings underpin the recently initiated phase I studies that are assessing the safety and antitumor activity of the bispecific T-cell engager KLK2 × CD3 (NCT04898634) and targeted radiotherapy ^225^Ac-KLK2 (NCT04644770).

## Introduction

Prostate cancer is one of the most common cancers among men worldwide, with an estimated 299,010 new cases diagnosed and 35,250 deaths expected from the disease in the United States in 2024 (1). The 5-year relative survival rate (2014–2020) for prostate cancer is 97.5% in the United States; however, for cases in which the cancer has metastasized at the time of diagnosis, the 5-year survival rate is 36.6% (2).

Androgen deprivation therapy (ADT), androgen receptor (AR) pathway inhibitors (ARPi), and chemotherapy are standard treatments for locally advanced or metastatic prostate cancer (3). In hormone-sensitive prostate cancer, ADTs, ARPis, and chemotherapy can achieve favorable clinical outcomes, but each of these treatment modalities has limitations that restrict their overall efficacy across a spectrum of patients with prostate cancer. The initial clinical response to ADTs or ARPis, which function by decreasing testosterone levels and attenuating AR signaling, can be temporary and can lead to castration-like symptoms (4). Additionally, prolonged exposure to ADT or ARPis can induce AR mutations and gene amplification, thus promoting disease progression by increasing AR signaling and selecting for prostate cancer cells resistant to further treatment with these agents (5). Although ADTs and ARPis are effective treatments, there is a need to develop hormone-sparing options not only to improve treatment safety and quality of life but also to relieve therapeutic pressure on AR signaling and delay disease progression toward castration-resistant phenotypes. Chemotherapy, added to ADT alone or in combination with ARPi, can be an effective alternative but comes at the price of systemic adverse events and a negative impact on quality of life (6–8). Expanding these options could help address the limitations associated with hormone-sensitive treatments and chemotherapy. Overall, this highlights the importance of identifying more effective therapies, including non-AR–targeted options, to treat a broader spectrum of patients with prostate cancer, particularly those with advanced prostate cancer or metastatic castration-resistant prostate cancer (mCRPC).

Prostate-specific membrane antigen (PSMA) is a well-established cell surface target in refractory/late-line mCRPC and a validated clinical target for the development of targeted therapies. These include radiopharmaceuticals (e.g., ^177^Lu-PSMA-617) and chimeric antigen receptor (CAR) T-cell therapies [e.g., autologous PSMA CAR T cells; US National Library of Medicine (cited June 17, 2025). Available from: https://clinicaltrials.gov]. However, current PSMA-targeted therapies have variable efficacy due to the inter- and intraheterogeneous expression of PSMA among patients (9, 10). Additionally, PSMA is expressed in a number of normal tissues, in particular the salivary and lacrimal glands, leading to potential on-target, off-tumor toxicities in non-prostatic tissues (10). Other prostate cell surface antigens, such as prostate stem cell antigen (PSCA) and six-transmembrane epithelial antigen of the prostate 1 (STEAP1), also exhibit nonspecific expression profiles, potentially leading to similar on-target, off-tumor effects (11–13).

Human kallikrein 2 (KLK2), encoded by the *KLK2* gene, is a secreted trypsin-like androgen-regulated serine protease produced by columnar prostate epithelial cells (14). KLK2 mediates semen liquefaction and increases the motility of spermatozoa during impregnation (15). KLK2 also activates prostate-specific antigen (PSA; or KLK3, encoded by the *KLK3* gene; ref. 16) and shares 80% amino acid homology with PSA (14). KLK2 and KLK3 are located approximately 12 kb apart on the q arm of chromosome 19 (17). Their close genomic proximity and functional interdependence likely contribute to the strong association observed between them at the protein level (18, 19). Previous reports showed that KLK2 expression is highly prostate-specific, with little-to-no expression in non-prostatic tissues (14, 20) and that its expression remains relatively homogeneous across disease stages in prostate cancer [Gene Page (cited June 17, 2025). Available from: http://gtexportal.org/home/gene/KLK2]. In addition, KLK2 levels correlate with disease severity (21, 22), and KLK2 has been reported to facilitate tumor growth and metastasis through several mechanisms, including activation of growth factor and mitogenic pathways (23–25). KLK2 is also regulated by androgen and AR signaling, and increased AR signaling activity has been associated with increased KLK2 and decreased PSMA levels (20, 26, 27). Thus, targeting KLK2 may provide more focused treatment outcomes, as reactivation of AR signaling is commonly observed in mCRPC (20, 26–28). However, due to the nature of being a serine protease, KLK2 is commonly characterized as a secreted soluble protein and deemed untargetable by biologics approaches due to a perceived lack of cell surface expression. Despite the increased interest in developing novel treatment options in mCRPC with multiple prostate-specific targets (e.g., PSMA, PSCA, and STEAP1), KLK2-targeting agents remain limited in the clinic.

In this study, we assessed the expression profile and cellular localization of KLK2 across disease stages of prostate cancer and characterized the therapeutic effect of three KLK2-targeting treatments in preclinical models of prostate cancer. Our results confirm that KLK2 is highly expressed across the prostate cancer disease continuum and characterize KLK2 as a promising cell surface therapeutic target in prostate cancer.

## Materials and Methods

### Ethics statement

All experiments were performed in accordance with the Guide for the Care and Use of Laboratory Animals, the Animal Welfare Act, and the Office of Laboratory Animal Welfare. They were approved by the Institutional Animal Care and Use Committee of Johnson & Johnson R&D. Male NOD/SCID gamma (NSG) mice (RRID:IMSR_JAX:005557; The Jackson Laboratory; T-cell engager and CAR T-cell evaluations) and athymic nude mice [RRID:IMSR_CRL:490; Charles River Laboratories; radioligand therapy (RLT) evaluation] were used at approximately 6 to 8 weeks of age. All animals were allowed to acclimate and recover from any shipping-related stress for a minimum of 5 days prior to experimental use. Healthy donor leukopaks were purchased from StemExpress between October 2021 and April 2022. Written informed consent was obtained, and leukapheresis was performed according to StemExpress Protocol 401-01 (WIRB#20152869).

### Tissue samples

Tissue samples for IHC/multiplex immunofluorescence (mIF) studies were sourced from the Prostate Cancer Biorepository Network (mCRPC) and Avaden Bio [localized prostate cancer (LPC) and metastatic hormone-sensitive prostate cancer (mHSPC)].

### Eukaryotic cell lines

The vertebral cancer of the prostate (VCaP; CRL-2876, RRID:CVCL_2235) and DU145 (HTB-81, RRID:CVCL_0105) cell lines were purchased from and authenticated by the ATCC and cultured according to the manufacturer’s instructions. Mycoplasma contamination of the VCaP and DU145 cell lines was also assessed by the ATCC.

### IHC

The deparaffinization, antigen retrieval, and immunostaining of samples were conducted on a Bond RX automated stainer (Leica; RRID:SCR_025548). Immunostained controls and samples were scanned on an Aperio AT2 scanner (Leica; RRID:SCR_021256) and reviewed for qualitative analysis by image capture. Tissue sections (4 μm) were placed on glass slides. Deparaffinization, epitope retrieval, and immunostaining of samples were conducted on the Leica Bond RX auto stainer. The default “Dewax” protocol in the Leica Bond RX auto stainer was utilized for the deparaffinization of slides, followed by Leica’s “H2(20)EDTA” protocol for heat-induced epitope retrieval. The following immunohistostaining procedure was conducted at room temperature (RT): 10 minutes of peroxidase blocking, protein blocks for 60 minutes, 3× washes, incubation with 0.2 μg/mL of KLK2 antibody for 60 minutes, 3× washes, application of post primary for 8 minutes, 3× washes, incubation of polymer for 10 minutes, 2× washes, rinsing with DI H20, incubation with mixed DAB refined for 10 minutes, 3× rinse with DI H20, counterstaining with hematoxylin for 5 minutes, and rinsing with DI H20. After immunostaining, slides were dehydrated through an alcohol series to absolute ethanol, followed by xylene rinses. Slides were permanently cover-slipped with glass coverslips and reviewed by a pathologist. Protein expression by IHC staining was visualized using a Leica Bond RX instrument with onboard antigen retrieval in ER2 solution. Where applicable, expression was quantified by *H* score based on stain intensity and tumor positivity. Rabbit monoclonal anti-human KLK2 antibody clone OT15D6 (OriGene; RRID:AB_2626200), anti-human CD4 antibody clone EPR6855 (Abcam; RRID:AB_2750883), anti-human CD8 antibody clone SP57 (Ventana; RRID:AB_2335985), and anti-human CK (AE1/AE3, Leica; RRID:AB_564123) were used for KLK2, CD4, and CD8 IHC.

### mIF

The mIF was used to assess KLK2 expression at different mCRPC tumor sites (160 total samples from 57 patients). The mIF assay iteratively stains, images, and performs advanced image analysis for marker expression at the single-cell level. Pathology review of the mCRPC samples confirmed the region of interest. KLK2 staining was required to be colocalized on epithelial origin cells by cytokeratin expression. Analysis of the position of staining relative to individual tumor cells was then extrapolated through image analysis.

For mIF assays, KLK2 samples were subsequently stripped of the primary antibody on a Bond RX, and the incubation process was repeated with clone 3E6 (Agilent, cat. # M3620, RRID:AB_2106450) to assess PSMA expression levels. Slides were digitally imaged, and fluorophore signals were unmixed on a PhenoImager HT scanner (Akoya Biosciences) to produce discrete signal localization. The tumor area was annotated, and intensity thresholds were set with pathologist's review using Akoya’s Phenoptics software. Cell segmentation, as well as phenotypic enumeration in the annotated areas, was performed using Akoya’s Proxima software. Pan-cytokeratin was utilized as the tumor mask to define which mCRPC cells to include in the analysis of the distribution and colocalization of KLK2 and PSMA. OT15D6 and PSMA antibody clone 3E6 (Agilent) were used for mIF assays.

### KLK2 FACS staining

KLK2 cell surface expression in fresh tumor samples of patients with mCRPC was assessed by flow cytometry using an anti-KLK2 antibody. Fresh mCRPC bone samples were obtained through an Institutional Review Board–approved research collaboration with Thomas Jefferson University. All patient samples were obtained under an approved Institutional Review Board protocol. All patients provided written informed consent for the use of research tissue; tissue samples were stored in a HIPAA-compliant manner, and all data were kept in a password-protected database. Tissue was resected and transferred fresh in RPMI media after surgery. Upon receipt of the tissue, the dissociation process using a human Tumor Dissociation Kit (Miltenyi, 130-095-929, RRID:SCR_020276) was performed as per the manufacturer’s protocol. Red blood cell lysis was done via the addition of ACK lysing buffer according to the manufacturer’s protocol. Once the tumor tissue was dissociated into single-cell suspensions, the FACS staining process was initiated. The cells were resuspended with 100 μL/well of LIVE/DEAD Blue stain diluted in Dulbecco’s PBS (DPBS; 1:1,000) and incubated for 15 minutes at RT in the dark. The cells were then washed twice with FACS stain buffer (BD Pharmingen Stain Buffer). An EpCAM (epithelial cell adhesion molecule) detecting antibody conjugated to BV421 (BioLegend, 324220, RRID:AB 2563847) was added to all wells and allowed to incubate for 30 minutes at 4°C. Cells were spun at 1,300 rpm for 3 minutes, and the supernatant was discarded. All wells were washed twice with FACS buffer at a volume of 200 μL. After each wash, cells were spun at 1,300 rpm for 3 minutes, and the supernatant was discarded. Next, the KLK2 detecting antibody conjugated to Alexa Fluor 647 was added to the wells at a 300 nmol/L concentration for 30 minutes at 4°C. After incubation, cells were spun at 1,300 rpm for 3 minutes, the supernatant was discarded, and two additional 200 μL washes were conducted. Finally, the detection of KLK2-positive tumor cells was conducted via flow cytometry on an LSRFortessa flow cytometer (BD Biosciences, RRID:SCR_018655) using FACSDiva Software (BD Biosciences, RRID:SCR_001456).

KLK2 cell surface expression in a human prostate cancer cell model (VCaP, ATCC, CRL-2876, RRID:CVCL_2235) was assessed by flow cytometry using an anti-KLK2 antibody. VCaP cells were dissociated with enzyme-free dissociation buffer (Thermo Fisher Scientific, 13150016). Cells were then aliquoted into 96-well, U-bottom plates, and a 100 μL PBS wash was added. Plates were spun at 1,300 rpm for 3 minutes, and the supernatant was removed. LIVE/DEAD Violet (Thermo Fisher Scientific, L34955) in PBS (1:3,000) was applied to the cells for 10 minutes at RT. The cells were then washed with FACS stain buffer (BD Pharmingen Stain Buffer). KLK2 antibody was added to the wells at 3 ng/μL for 30 minutes at RT. After incubation, cells were spun at 1,300 rpm for 3 minutes, the supernatant was discarded, and two additional 200 μL washes were conducted. Alexa Fluor 647 AffiniPure Donkey Anti-Human IgG (3 μg/mL; Jackson ImmunoResearch) was added to cells for 30 minutes at RT. Cells were washed two times with stain buffer. Detection of KLK2-positive tumor cells was conducted via flow cytometry on a BD FACSCelesta Flow Cytometer (BD Biosciences, RRID:SCR_019597) and analyzed using FACSDiva Software (BD Biosciences, RRID:SCR_001456).

### Confocal imaging

KLK2-targeting antibody internalization over time was visualized by confocal IF microscopy in VCaP cells. VCaP cells (acquired from ATCC, CRL-2876, RRID:CVCL_2235) were cultured in DMEM, low glucose, GlutaMAX supplement, and pyruvate (Gibco, 10567-014) and supplemented with 15% heat-inactivated FBS (Gibco, 16140071). VCaP cells (10,000 cells per well in 40 μL) were plated on a 384-well poly-D-lysine–coated plate (Revvity, 6057500) and processed 48 hours after plating. Cells were fixed in 3% paraformaldehyde (Electron Microscopy Sciences, 15714) in DPBS (Gibco, 14190144) for 20 minutes at RT and then washed with DPBS. Cells were blocked in 5% goat serum (Sigma, G9023-5ML) in DPBS for 1 hour at RT.

KLK2 (KLK2 × CD3) or isotype (anti–respiratory syncytial virus in place of KLK2 binding arm) antibody staining solutions (20 nmol/L) in 5% goat serum in DPBS were prepared and applied to cells for 1 hour at RT. Cells were washed with DPBS, and then a 2 μg/mL secondary staining solution in 5% goat serum in DPBS was applied (Thermo Fisher Scientific, A11013) for 1 hour at RT. Cells were then washed with DPBS. The Hoechst (Thermo Fisher Scientific, H3569; 2 μg/mL) and EpCAM (BioLegend, 324212; 1:50, RRID:AB_756086) staining solutions were prepared in 5% goat serum in DPBS and applied for 30 minutes at RT. Cells were then washed with DPBS, sealed, and stored at 4°C until imaging. Cells were imaged on the Opera Phenix (Revvity, RRID:SCR_021100), and images were analyzed using Fiji software (RRID:SCR_002285). Anti-KLK2 × anti-CD3 (KLK2 × CD3) was used for confocal imaging, *in vitro* studies, and *in vivo* studies. Anti–Rous sarcoma virus × anti-CD3 (isotype) was used for confocal imaging. Anti-KLK2 ^225^Ac-DOTA-h11B6 was used for *in vivo* studies.

### Visualization of immune synapses

VCaP cells acquired from the ATCC (CRL-2876, RRID:CVCL_2235) were transduced with IncuCyte NucLight Red Lentivirus (NLR; EF1a and Bleo; Sartorius, item # 4478) to produce VCaP-NLR cells. VCaP-NLR cells were trypsinized, neutralized with supplemented media [DMEM, low glucose, GlutaMAX supplement, and pyruvate (10567-014) + 15% heat-inactivated FBS], washed with supplemented media, and then counted using a Vi-CELL BLU instrument (Beckman Coulter, RRID:SCR_026900). VCaP-NLR cells were then incubated with LIVE/DEAD Near-IR (Thermo Fisher Scientific, L34975; 1:1,000) in DPBS for 10 minutes. Leukopaks from healthy male donors were purchased from StemExpress and shipped at RT prior to processing and T-cell isolation. T cells were isolated using the Pan T Cell isolation kit (Miltenyi, 130-096-535) on the CliniMACS Prodigy system (Miltenyi, RRID:SCR_020289). Cells were resuspended in CryoStor-10 (Biolife Solutions, 210102), aliquoted, frozen, and stored in liquid nitrogen until used in assays. Five healthy male T-cell donors were thawed, washed with supplemented media, counted, and then incubated with 0.5 μmol/L CellTrace Violet dye (Thermo Fisher Scientific, C34571) and LIVE/DEAD Near-IR in DPBS for 10 minutes. After the CellTrace and LIVE/DEAD incubations, cells were washed with supplemented media.

T cells and VCaP-NLR cells were combined in a 96-well plate at a ratio of 2:1, respectively, for 24 hours at 37°C with 5% CO_2_ with or without 30 nmol/L KLK2 × CD3 (JNJ-78278343) or null × CD3. Cells were fixed in 4% paraformaldehyde (Electron Microscopy Sciences, 15714) for 15 minutes, washed gently, then permeabilized with a solution of 0.1% Triton and 1% BSA in stain buffer (BD Biosciences, 554656) with phalloidin (AF488, A12379) and Alexa Fluor 647 secondary antibody (Donkey Anti-human IgG H+L, cat. # 709-605-149, RRID:AB_2340578) to visualize the KLK2 × CD3 or null × CD3 for 30 minutes at RT, and then washed with stain buffer. Samples were run in 40 μL stain buffer on the ImageStream Mk II Imaging Flow Cytometer (Cytek Biosciences, RRID:SCR_018589). All wash steps were performed with centrifugation at 1,200 rpm for 5 minutes at RT.

### Mice xenograft models to assess antitumor activity of KLK2-targeting agents

For all experiments, VCaP (ATCC, CRL-2876, RRID:CVCL_2235) xenografts were used to evaluate antitumor activity, defined as the difference between the mean tumor volume of the treatment and control groups, corrected for initial tumor burden [% Δtumor growth inhibition (TGI)]. To generate VCaP xenografts, 1 × 10^7^ cells (in 50% Cultrex or Matrigel) were injected subcutaneously in the right flank on day 0. Tumor volume and body weight were measured twice weekly and graphed using GraphPad Prism (version 8, RRID:SCR_002798).

#### KLK2 × CD3 *in vivo* efficacy

To humanize the NSG mice, human Pan-T cells (AllCells) were activated and expanded *in vitro* using the T-cell activation and expansion kit and grown in medium containing IL-2 at a concentration of 0.1 μg/μL, starting 3 days after thawing. On the day of engraftment into mice, beads were removed from the T cells using magnets, and the cells were resuspended in serum-free medium for an i.p. injection of 2 × 10^7^ cells in 0.2 mL per mouse. T cell–engrafted mice were given fragment crystallizable block at 0.2 mg/mouse intraperitoneally and human immune globulin infusion at 10 mg/mouse intraperitoneally at least an hour prior to antibody dosing to compensate for the low immunoglobulin environment in the NSG mouse.

On day 20 after s.c. VCaP (ATCC, CRL-2876, RRID:CVCL_2235) cell implantation, animals were randomized into groups of 10 by tumor volume and engrafted with human T cells the next day. Treatment was initiated 1 day later with i.p. dosing twice a week of KLK2 × CD3 (0.2, 1, 2.5, 5, or 15 mg/kg) or null × CD3 antibody at 15 mg/kg for a total of eight doses. At the end of the study, tumors from the null × CD3 control and KLK2 × CD3 15 mg/kg treatment groups were collected, fixed in 10% neutral buffered formalin for approximately 48 hours, and then stored in 70% ethanol, sectioned, and stained for tumor and immune effector cell markers.

#### KLK2 RLT *in vivo* evaluation

Treatment with a single dose of actinium-225 (^225^Ac)–labeled KLK2-targeting antibody (50, 100, 250, or 500 nCi) or vehicle was injected via the tail vein after s.c. VCaP (ATCC, CRL-2876 RRID:CVCL_2235) tumors were established, and animals were randomized (day 22 after VCaP cell implantation) into groups of 10 by tumor volume.

#### KLK2 CAR T-cell *in vivo* evaluation

On day 21 after s.c. VCaP (ATCC, CRL-2876, RRID:CVCL_2235) cell implantation, animals were randomized into groups of 10 by tumor volume. The next day, animals were given a single i.v. dose of KLK2 CAR T cells (1 × 10^6^, 5 × 10^6^, or 10 × 10^6^ CAR T cells), mock untransduced T cells, or serum-free RPMI medium. Peripheral blood samples were collected via the retro-orbital route from five animals per group for immunophenotyping by flow cytometry on days 7, 14, 29, and 36 after KLK2 CAR T-cell treatment for immunophenotyping, assessment of CAR T-cell expansion, and assessment of circulating IFN-γ.

### Cell binding assay

VCaP (ATCC, CRL-2876, RRID:CVCL_2235) and DU145 (ATCC, HTB-81, RRID:CVCL_0105) cells were washed with DPBS and harvested with 5 mL TrypLE. The dissociated cells were then collected and centrifuged at 1,300 rpm for 3 minutes. The cell pellet was washed, resuspended in 1 mL DPBS, and counted using the Vi-CELL XR cell viability analyzer (Beckman Coulter, RRID:SCR_019664). Next, 100,000 cells per 100 μL of DPBS were added to the wells of a clear 96-well, U-bottom plate. The plate was spun at 1,350 rpm for 3 minutes, and the supernatant was discarded. The cells were resuspended with 100 μL/well of LIVE/DEAD Blue stain diluted in DPBS (1:1,000) and incubated for 15 minutes at RT in the dark. Next, the cells were washed twice with FACS stain buffer (BD Pharmingen Stain Buffer). The test antibodies (KLK2 × CD3 and isotype control) were diluted to a starting concentration of 6 μmol/L in stain buffer, and serial dilutions were prepared from the starting concentration with threefold dilution to obtain a total of 12 concentration points down to 0.03 nmol/L. The serially diluted test antibodies (100 μL/well) were added, and the plates were incubated for 45 minutes at 37°C in the dark. The cells were centrifuged, pelleted, and washed twice with stain buffer, followed by resuspension in 100 μL/well of 1:200 diluted anti-human IgG heavy + light chain secondary antibody (The Jackson Laboratory, 709-605-149, RRID:AB_2340578) and then incubated for 30 minutes at 4°C in the dark. The cells were washed twice with stain buffer and resuspended in 100 μL of stain buffer prior to analysis on an LSRFortessa flow cytometer (BD Biosciences, RRID:SCR_018655) using FACSDiva Software (BD Biosciences, RRID:SCR_001456). Results for the binding of the test antibodies to VCaP and DU145 cells were analyzed in FlowJo software (BD Biosciences, version 10, RRID:SCR_008520). The geometric mean fluorescence intensity values for the test antibodies were graphed in Prism (GraphPad, version 8, RRID:SCR_002798). The data were log_10_-transformed and fit to a log(agonist) versus response variable slope (four-parameter) nonlinear equation.

#### Immunophenotyping of whole blood from mice treated with KLK CAR T Cells

On days 7, 14, 29, and 36 and after KLK2 CAR T-cell treatment, peripheral blood samples were collected in Microtainer blood collection tubes via retro-orbital or cardiac puncture routes from mice. Blood samples (100 μL) were transferred into 96-well, U-bottom plates and centrifuged at 1,000 × gravity (*g*) for 10 minutes at 4°C. Plasma was collected and stored at −80°C immediately after spin until cytokine analysis was performed. Red blood cells in the remaining cells were lysed using 100 µL ACK lysis buffer for 3 to 5 times until no more red debris could be seen. Cells were pelleted, washed in 200 μL DPBS twice, and reconstituted in 100 μL stain buffer. LIVE/DEAD staining was carried out with 1 μg/mL KLK2-biotin antigen for 15 minutes at RT. Cells were washed and centrifuged at 300 × *g* 3 times. A directly conjugated antibody cocktail (CD3, CD4, CD8, anti-biotin antibodies) was added at the manufacturer’s recommended volume, and cells were stained for 30 minutes at 4°C. Cells were washed, pelleted with stain buffer 3 times, and reconstituted in 100 μL stain buffer before acquisition on an LSRFortessa flow cytometer (BD Biosciences, RRID:SCR_018655). CAR^+^ events were recorded from the LIVE/CD3^+^ cell gate. The absolute number of CAR^+^ events was used to calculate the number of CAR^+^ cells per 100 μL blood.

#### IHC staining of T-cell infiltration

The CD8 IHC staining was performed on a Discovery XT system (Ventana; RRID:SCR_026335). Antigen retrieval was conducted with cell conditioning 1 at 95°C for 8 minutes and 100°C for 4 minutes. Sections were treated with 4% H_2_O_2_ to block endogenous peroxidases, followed by incubation with the primary anti-CD8 antibody (RRID:AB_2335985; 0.35 μg/mL) at RT for 60 minutes. After rinsing, an antibody block was applied for 16 minutes, followed by the OmniMap anti-Rb HRP secondary antibody for 28 minutes. Detection was performed using the ChromoMap DAB Detection Kit, with hematoxylin and bluing counterstaining (8 minutes each) before cover-slipping.

CD4, KLK2, and cytokeratin IHC staining were conducted on a Bond RX automated stainer (Leica; RRID:SCR_025548). For CD4 and KLK2, antigen retrieval was performed with Bond Epitope Retrieval Solution 2 at 100°C for 20 minutes, whereas cytokeratin used Bond Epitope Retrieval Solution 1 at 100°C for 10 minutes. Sections were treated with 3% H_2_O_2_ (KLK2 and cytokeratin) and protein-blocked (60 minutes for KLK2, 15 minutes for cytokeratin). Primary antibody incubation was carried out at RT for 60 minutes for CD4 (1:100 dilution) and KLK2 (0.2 μg/mL mouse monoclonal) and for 30 minutes for cytokeratin. Detection and counterstaining for CD4, KLK2, and cytokeratin were performed using the Bond Polymer Refine Detection Kit, with hematoxylin counterstaining (5 minutes) before cover-slipping. Representative images of stained tumors were taken.

### *In vitro* pharmacology assays (cytotoxicity, T-cell activation, and cytokine release)

VCaP (ATCC, RL2876, RRID:CVCL_2235) cells were plated in 96-well, flat, clear-bottom, black plates 48 hours before the addition of drug and peripheral blood mononuclear cells (PBMC), to enable attachment. DU145 (ATCC, HTB-81, RRID:CVCL_0105) cells were plated in the same type of plates 24 hours prior to the addition of drug and PBMCs, based on faster adherence than VCaP cells. Target cells were washed with DPBS, harvested with TrypLE cell dissociation buffer, and washed once with phenol red–free complete medium. Frozen vials of five healthy male donor PBMCs (procured from Discovery Life Sciences, Inc.) were thawed in a 37°C water bath, transferred to 15 mL conical tubes, and washed once with 5 mL phenol red–free complete medium. The cells were centrifuged at 500 × *g* for 5 minutes, pelleted, resuspended in 1 mL of phenol red–free complete medium, and counted using the Vi-CELL XR cell viability analyzer (Beckman Coulter, RRID:SCR_019664). Next, PBMCs were added to the target cells at a predefined effector/target ratios normalized by the percentage of CD3^+^ T cells from each donor’s PBMCs. The test antibodies (KLK2 × CD3 or null × CD3 control) were diluted to a final starting concentration of 15 μg/mL in phenol red–free complete medium, and threefold serial dilutions were prepared from the starting concentration for a total of 10 dilution points. Assays were run for up to 96 hours. Cytotoxicity was assessed as described below. T cells and supernatants were harvested at different time points to measure T-cell activation and cytokine secretion, respectively.

Target cell cytotoxicity was assessed from some redirection assays. The plates were placed in the IncuCyte S3 (Sartorius, RRID:SCR_023147) at 37°C with 5% CO_2_ for up to 96 hours. Red object counts (indicating viable target cells) per well were measured by the IncuCyte S3. Data were directly exported using the IncuCyte S3 software, in which red object counts (number of viable target cells) were determined for each well and used to calculate the percentage of lysis. Concentration–response curves were graphed in Prism (GraphPad, RRID:SCR_002798), in which the relative percentage of tumor cell growth inhibition was plotted along the *Y*-axis, and the concentration of the test antibodies was plotted along the *X*-axis. Antibody concentrations were log_10_-transformed, and data were fit to a log(agonist) versus response variable slope (four-parameter) nonlinear equation.

For T-cell activation, plates from the cytotoxicity assay were centrifuged at 1,300 rpm for 3 minutes. Clarified supernatant (approximately 100 μL) was transferred to a clear 96-well, U-bottom plate for the detection of T-cell-mediated cytokine release. The cell mixture was transferred to a new clear 96-well, U-bottom plate and spun at 350 × *g* for 3 minutes, the supernatant was discarded, and 100 μL of DPBS was added. The cells were stained with LIVE/DEAD Blue by adding 100 μL of the working solution (prepared by adding 50 μL of DMSO to the lyophilized vial and then diluting the stock solution 1:1,000 in DPBS) and incubated for 15 minutes at RT in the dark. Plates were centrifuged at 1,300 rpm for 3 minutes at RT, and the supernatants were discarded. Cell pellets were washed twice with 100 μL of stain buffer, resuspended with 100 μL/well of the staining antibody panel, and incubated for 30 minutes at 4°C in the dark. Finally, the cells were washed twice and resuspended in 100 μL of stain buffer prior to analysis on an LSRFortessa flow cytometer (BD Biosciences, RRID:SCR_018655) using FACSDiva Software (BD Biosciences, RRID:SCR_001456).

The antibody panel included anti–hCD3-BV711 (clone SK7; BD Biosciences, 740832, RRID:AB_2740489; dilution 1:50), anti–hCD25-APC (clone MA-251; BD Biosciences, 555434, RRID:AB_398598; dilution 1:5), and anti–hCD69-PE (clone FN50; BioLegend, 310906, RRID:AB_314841; dilution 1:50).

T-cell activation results for the test antibodies were analyzed in FlowJo (BD Biosciences, version 10, RRID:SCR_008520), in which the following gating strategy was applied: cell population [forward scatter (FSC)-area × side scatter (SSC)-area], single cells (FSC-area × FSC-height), live cells (histogram with live events), CD3^+^ cells (histogram with CD3^+^ events), and then CD25^+^ or CD69^+^ cells (histogram with CD25^+^/CD69^+^ events). The percentage of CD25^+^ or CD69^+^ cells (activated T cells) within the CD3^+^ live population was graphed using Prism version 8 (GraphPad, RRID:SCR_002798).

For cytokine analysis, supernatants from the T-cell redirection assays were harvested and stored at −80°C until analyzed using the MILLIPLEX MAP Human 13-plex multiplex (Millipore, RRID: AB_3695989) assay according to the manufacturer’s protocol. The kit measures the following cytokines: IFN-γ, IL-1β, IL-2, IL-4, IL-5, IL-6, IL-7, IL-8, IL-10, IL-12(p70), IL-13, GM-CSF, and TNF-α. Supernatants were thawed on wet ice, spun at 1,300 rpm for 5 minutes at 4°C, and then placed on ice. The plate preparation and assay were conducted according to the manufacturer’s protocol without any modification. Assay plates were read on the FLEXMAP 3D instrument (Luminex, RRID:SCR_026299). GM-CSF, IFN-γ, and TNF-α levels were used to confirm T-cell activation and represented Th1 T-cell functional cytokine release.

### Statistical analysis

Graphical data are presented as the mean ± SEM unless otherwise indicated. Differences between the groups were tested using a two-tailed unpaired Student *t* test, using GraphPad Prism 9 (RRID:SCR_002798).

## Results

### KLK2 expression profile in prostate cancer

In contrast to the expression of well-established prostate cancer targets (PSMA, PSCA, and STEAP1), KLK2 expression is highly prostate-specific (21, 22). To further demonstrate the specific expression profile of KLK2, we analyzed the mRNA expression of KLK2, PSMA, and STEAP1 from a publicly available expression dataset (29).

**Figure 1.**
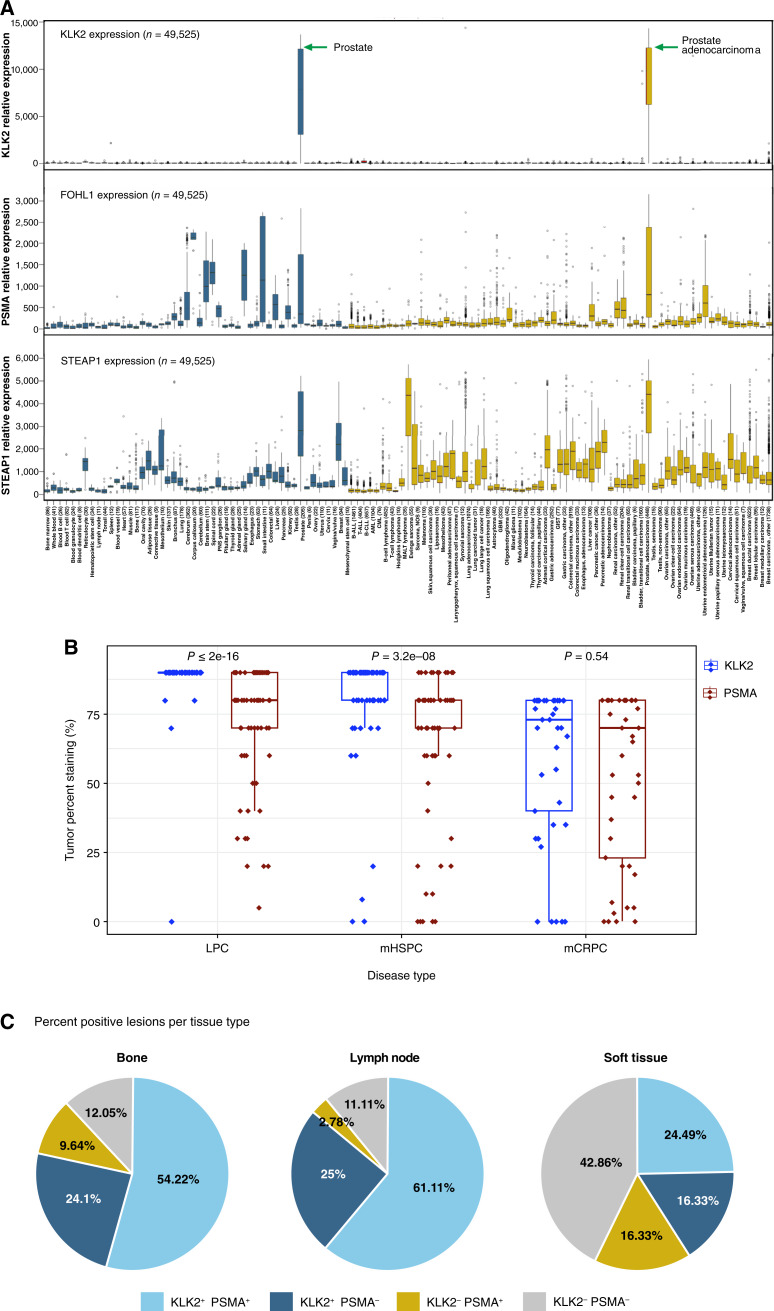
KLK2 expression profile. **A,** Box plot analysis of the KLK2, PSMA, and STEAP1 relative expression in normal human tissue types (*n* = 43) and cancer types (*n* = 68). The box refers to the quartile distribution (25%–75%) range, with the median shown as a black horizontal line. In addition, the 95% range and individual outlier samples are shown. **B,** KLK2 and PSMA protein expression in different stages of prostate cancer [LPC (*n* = 100), mHSPC (*n* = 100), and mCRPC (*n* = 45)] by IHC with semiquantitative pathologist reporting of the percentage of tumor showing any staining. The box refers to the IQR (25%–75% of samples), with the median shown as a horizontal line in the middle. In addition, whisker length extends to 1.5 × IQR on both sides of the IQR, and individual outlier samples are shown. **C,** Proportions of bone, lymph node, and soft tissue lesions showing coexpression of KLK2 and PSMA, as evaluated by mIF. The lesion is considered positive when ≥50% of cells are stained positive by mIF. AML, acute myeloid leukemia; B-ALL, B-cell acute lymphoblastic leukemia; B-CLL, B-cell chronic lymphocytic leukemia; CML, chronic myeloid leukemia; GBM, glioblastoma multiforme; GIST, gastrointestinal stromal tumor; MALT, mucosa-associated lymphoid tissue; NOS, not otherwise specified; PNS, peripheral nervous system; T-ALL, T-cell acute lymphoblastic leukemia.

Consistent with previous reports, KLK2 expression was restricted to normal prostatic tissues and prostate adenocarcinoma with little-to-no expression outside the prostate (Fig. 1A). The expression of PSMA and STEAP1 was less specific, with broader expression across multiple non-prostate tissue sites (Fig. 1A).

To further characterize the KLK2 expression in prostate cancer, we analyzed protein level expression of KLK2 in LPC, mHSPC, and mCRPC tissue samples by IHC and mIF. Robust expression of KLK2 was noted across the prostate cancer disease continuum, with homogeneous expression in LPC and mHSPC and increased heterogeneity in heavily pretreated mCRPC tissue samples (Fig. 1B; Supplementary Fig. S1). Strong KLK2 IHC staining (intensity score = 3) was observed in the majority of cases of LPC (91/100), mHSPC (81/98), and mCRPC (34/45; Fig. 1B). Additionally, analysis of KLK2 expression in paired bone lesions within the same patient showed a high level of correlation (R = 0.74, *P* < 1.6e–06), suggesting relative intrapatient and interlesional homogeneity of KLK2 expression in bone lesions (Supplementary Fig. S2).

To further characterize the expression profile of KLK2 in prostate cancer, we compared its expression patterns with those of PSMA in the same tumor samples. These IHC data revealed that KLK2 expression was more homogeneous than that of PSMA in hormone-sensitive disease (LPC and mHSPC), in which KLK2 was almost universally expressed. In heavily pretreated mCRPC (rapid autopsy), KLK2 and PSMA showed similar heterogeneous expression profiles (Fig. 1B).

To enable a direct and quantitative comparison between KLK2 and PSMA expression at the lesional level, we set an arbitrary definition for lesion positivity as ≥50% of positive cells based on mIF imaging analysis. Our results showed that KLK2 was coexpressed with PSMA in bone (54.2% double-positive lesions), lymph node (61.1% double-positive lesions), and soft-tissue metastasis samples (24.5% double-positive lesions) from patients with mCRPC (Fig. 1C; Supplementary Fig. S3A and S3B). Trends of higher positivity rates of KLK2 than that of PSMA in lymph nodes [86.1% (confidence interval, or CI, 69.7–94.8) vs. 63.9% (CI, 46.2–78.7) positive lesions] and bone metastases [78.3% (CI, 67.6–86.3) vs. 63.9% (CI, 52.5–73.9) positive lesions], whereas the soft-tissue positivity rates were similar [40.8% (CI, 52.5–73.9) vs. 40.8% (CI, 27.3–55.7) lesions positive for KLK2 and PSMA, respectively; Fig. 1C]. In total, 83 bone, 36 lymph node, and 49 soft tissue samples were used in the analysis.

### KLK2 is expressed on the surface of prostate cancer cells

**Figure 2.**
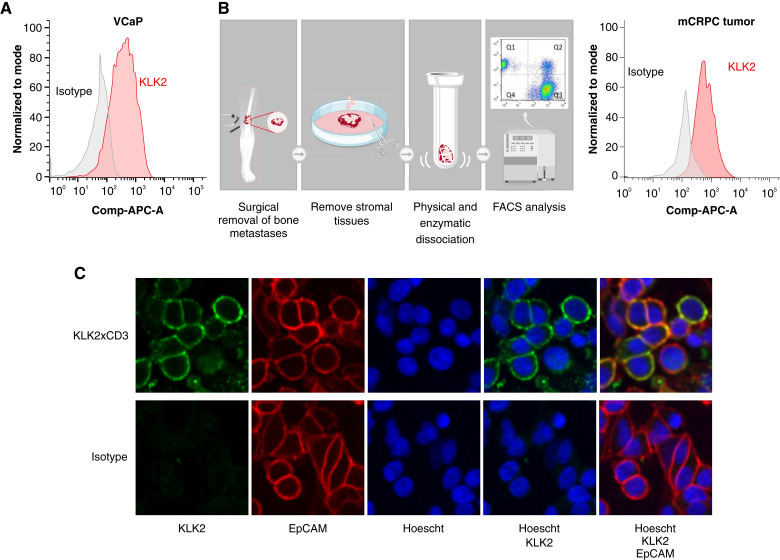
Cell surface expression of KLK2. **A,** FACS staining of KLK2 in VCaP cells. A representative FACS plot from *n* = 3 repeated experiments is shown. **B,** Workflow and FACS staining of KLK2 in freshly dissociated mCRPC tumor cells. Representative FACS plot is shown; *n* = 3. **C,** Confocal imaging of KLK2 surface expression in VCaP cells. A representative IF images are shown from *n* = 3 repeated experiments: KLK2 stain (green), EpCAM cell surface stain (red), and Hoechst DNA stain (blue).

KLK2 had previously been characterized as a secreted serine protease without evidence for cell surface expression (14, 20). To reassess potential cell surface expression, we used an anti-KLK2 monoclonal antibody to test a prostate cancer cell line (VCaP) as well as dissociated bone metastases from patients with mCRPC. Cell surface expression of KLK2 was confirmed by FACS of VCaP cells (Fig. 2A) and dissociated mCRPC tumor cells (Fig. 2B). Additionally, KLK2 cell surface expression in VCaP cells was visualized by confocal microscopy. The fluorescence signal of the anti-KLK2 antibody colocalized with the cell surface marker EpCAM, further confirming the cell surface expression of KLK2 (Fig. 2C).

### Targeting KLK2 with a bispecific T-cell engager induces T-cell activation and tumor cytotoxicity

**Figure 3.**
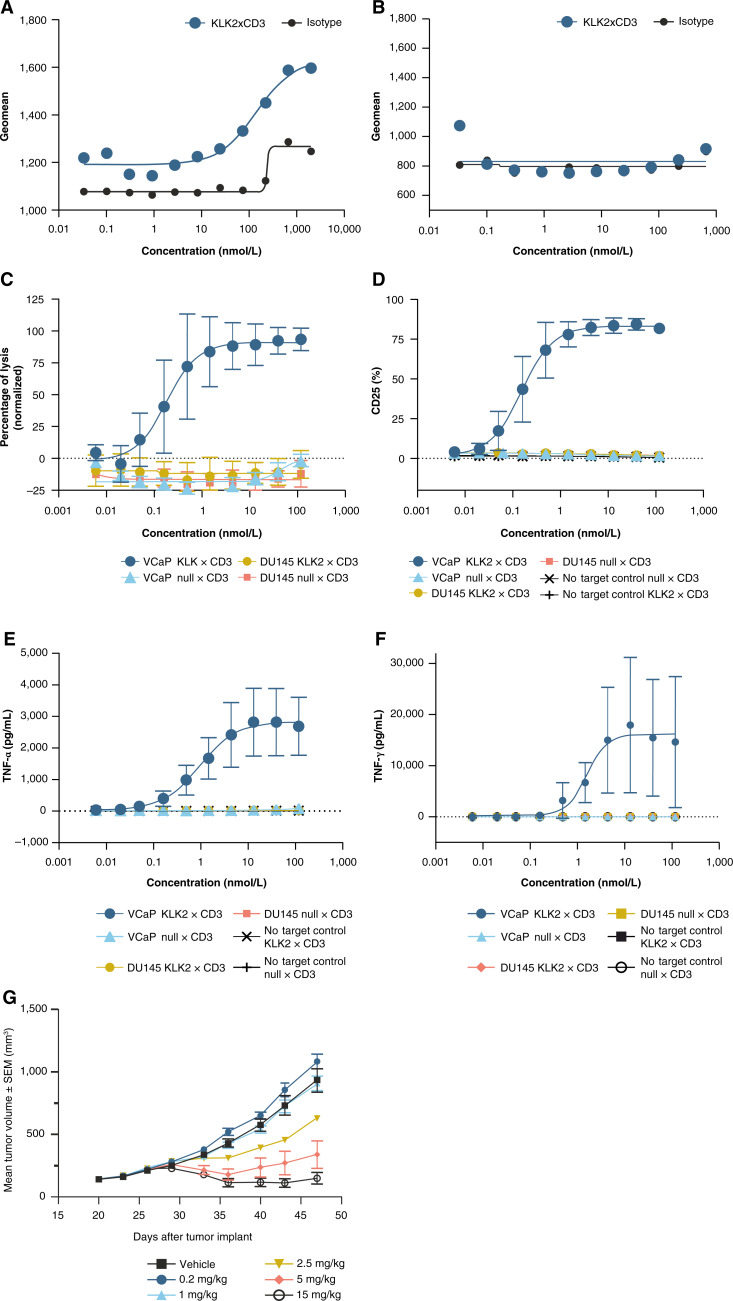
*In vitro* and *in vivo* characterization of KLK2 × CD3 in preclinical models. **A** and **B,** Binding of KLK2 × CD3 to (**A**) VCaP (KLK2^+^) and (**B**) DU145 (KLK2^−^) cells. A representative dose–response binding curve is shown; *n* = 5. **C–F,** Evaluation of KLK2 × CD3 on **(C)** T cell–mediated cytotoxicity, (**D**) T-cell activation, and (**E** and **F**) TNF-α and IFN-γ cytokine release *in vitro*, in PBMC from five healthy donors. Mean values ± SEM are shown. *n* = 5. **G,** T cell–engrafted NSG mice bearing established VCaP tumors were intraperitoneally dosed with KLK2 × CD3 at 0.2 mg/kg, 1 mg/kg, 2.5 mg/kg, 5 mg/kg, and 15 mg/kg, compared with control (eight total doses). Tumor volume was measured twice weekly, and the results are presented as the mean tumor volume ± SEM for each group (*n* = 8 per group).

**Figure 4.**
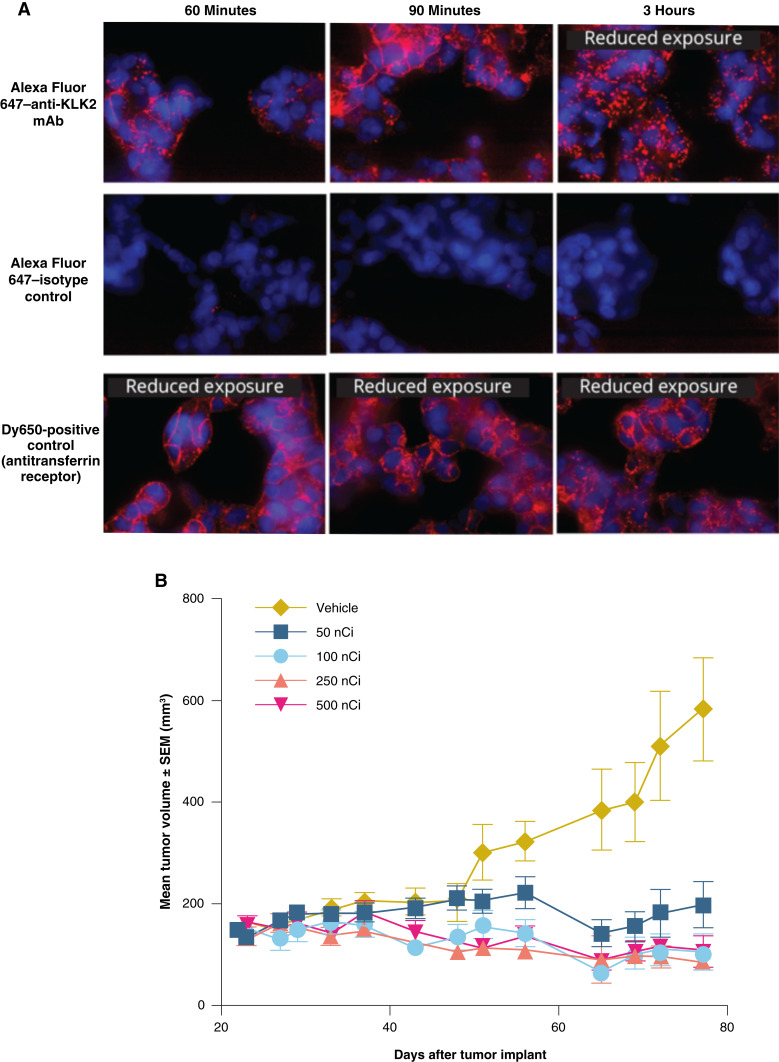
Preclinical characterization of KLK2-targeted RLT. **A,** Cell binding and internalization of the anti-KLK2 antibody in VCaP cells assessed by confocal imaging. Cell surface binding of the anti-KLK2 antibody was detected at 60 minutes with intracellular puncta formation observed at 3 hours, indicating antibody internalization. A representative confocal image is shown; *n* = 3. **B,** Treatment with a single i.v. dose of ^225^Ac-KLK2 in an established prostate xenograft model at 50, 100, 250, and 500 nCi, compared with control. Tumor volume was measured twice weekly, and the results are presented as the mean tumor volume ± SEM for each group (*n* = 10 per group).

To evaluate KLK2 as a potential therapeutic target for T-cell engagers, we developed a bispecific antibody, KLK2 × CD3, which binds to CD3 receptor complexes on T cells and KLK2 on the surface of prostate tumor cells. The formation of an immune synapse via this bispecific antibody is intended to activate T cells by binding to CD3 and redirecting them to KLK2-positive prostate cancer cells, facilitating KLK2-specific T-cell activation and targeted tumor lysis. In cell binding studies, KLK2 × CD3 bound to KLK2^+^ VCaP cells in a concentration-dependent manner, and no binding was observed with KLK2^−^ DU145 cells (Fig. 3A and B), demonstrating the target specificity of the bispecific antibody. The introduction of KLK2 × CD3 increased interactions between prostate cancer target cells (VCaP) and T cells from healthy male donors 1.5× and 2× compared with a null × CD3 control construct or with coincubation with no bispecific antibody, respectively (Supplementary Fig. S4A). KLK2 × CD3–mediated immune synapse formation was observed by F-actin polarization with KLK2 staining on VCaP cells and CD3 staining on T cells (Supplementary Fig. S4B), indicating increased engagement of T cells and prostate cancer cells in the presence of KLK2 × CD3. To characterize the pharmacologic activity of KLK2 × CD3, KLK2^+^ VCaP cells were treated with a wide dose range of KLK2 × CD3 in the presence of PBMCs from healthy male donors. At a 3:1 effector-to-target cell ratio, KLK2 × CD3 induced potent and dose-dependent T cell–mediated cytotoxicity, T-cell activation, and proinflammatory cytokine release, whereas the null × CD3 control antibody demonstrated minimal-to-no effects (Fig. 4C–F).

Next, we evaluated the *in vivo* antitumor activity of KLK2 × CD3 in a mouse xenograft model of prostate cancer. Consistent with our *in vitro* data, KLK2 × CD3 demonstrated dose-dependent TGI. *In vivo* efficacy was observed at doses as low as 2.5 mg/kg with more than 100% TGI reached at 15 mg/kg (Fig. 3G). To confirm the putative mechanism of action, on-treatment tumor samples were collected at several time points for IHC staining of selected tumor and T-cell markers. Representative micrographs of CD8, CD4, KLK2, and pan-cytokeratin immunostaining of tumor samples collected at day 50 (end of study) from the null × CD3 and KLK2 × CD3 15 mg/kg treatment groups are shown. As compared with VCaP tumors from null × CD3–treated mice, KLK2 × CD3 treatment resulted in increased CD8^+^ and CD4^+^ T-cell infiltration, with a concomitant reduction of KLK2+ tumor cells as measured by the detection of KLK2 and cytokeratin. Taken together, these studies validate KLK2 as a surface target in prostate cancer and a KLK2 × CD3 T-cell engager as a potential therapeutic treatment (Supplementary Fig. S5).

### KLK2-targeted radioconjugate (^225^Ac-KLK2) exhibits target-specific uptake and antitumor activity in preclinical models

To determine whether KLK2 is amenable to additional targeting modalities, we next investigated KLK2 as a potential target for RLT. Given the notion that RLT agents are generally effective when internalized, we first tested for KLK2 internalization by labeling the anti-KLK2 monoclonal antibody with Alexa Fluor 647 to visualize the dynamics of KLK2 localization upon antibody binding. An anti–transferrin receptor antibody was used as a positive control for internalization. Multicolor confocal microscopy revealed significant cell surface binding of the anti-KLK2 antibody at 60 minutes, with increased surface signals at 90 minutes after antibody incubation. Notably, intracellular puncta formation was detected at 3 hours, indicating antibody internalization (Fig. 4A). No surface or intracellular signal was detected with the null control. As expected, the positive-control anti–transferrin receptor antibody also showed strong surface binding and rapid internalization. Next, the anti-KLK2 monoclonal antibody was conjugated to the chelator DOTA and subsequently radiolabeled with the α emitter ^225^Ac. To evaluate the *in vivo* antitumor activity of ^225^Ac-KLK2, multiple dose levels of ^225^Ac-KLK2 were investigated in the VCaP xenograft model. Treatment of VCaP tumor–bearing mice with a single i.v. injection of ^225^Ac-KLK2 reduced tumor growth across multiple doses, with 110% TGI achieved at 500 nCi compared with control (Fig. 4B).

### KLK2-targeted CAR T cells demonstrate robust antitumor activity in a xenograft model

**Figure 5.**
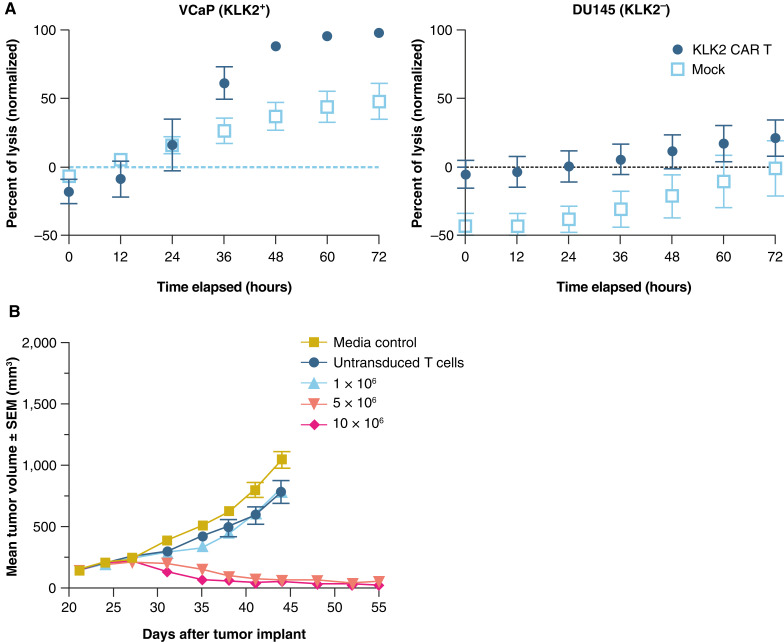
Preclinical characterization of KLK2-targeted CAR T cells. **A,** KLK2-targeted CAR T cells exhibit cytotoxicity when cocultured with KLK2^+^ but not with KLK2^–^ prostate cancer cell lines. The mean percentage of tumor lysis ± SEM is shown. A representative dose–response curve is shown; *n* = 3. **B, ***In vivo* i.v. treatment with KLK2-targeted CAR T cells (1 × 10^6^, 5 × 10^6^, and 10 × 10^6^ CAR T cells) on established VCaP human prostate xenografts. Tumor volume was measured twice weekly, and the results are presented as the mean tumor volume ± SEM for each group (*n* = 10 per group).

To evaluate a third KLK2-based treatment modality, autologous CAR T cells were generated using healthy donor T cells and engineered to target KLK2 via a scFv-based CAR (Supplementary Fig. S6). In coculture, KLK2 CAR T cells exhibited cytotoxicity against KLK2^+^ VCaP cells, but not KLK2^−^ DU145 cells (Fig. 5A). As expected, untransduced T cells did not induce significant target killing in either KLK2^+^ or KLK2^−^ cell lines. Additionally, in VCaP tumor–bearing mice, a single i.v. treatment with KLK2 CAR T cells resulted in robust TGI as compared with both the vehicle control–treated group and the untransduced control T-cell group, with 7/10 and 10/10 complete tumor regressions after infusion of 5 × 10^6^ and 10 × 10^6^ KLK2 CAR T^+^ cells, respectively (Fig. 5B). Tumor regression was accompanied by KLK2 CAR T-cell expansion and cytokine release, consistent with the functionality of KLK2 CAR T cells after tumor antigen exposure *in vivo* (Supplementary Fig. S7A). Mechanistically, we observed robust CD4^+^ and CD8^+^ T-cell infiltration into tumors after 14 days of treatment with KLK2 CAR T^+^ cells in both 5 × 10^6^ and 10 × 10^6^ treatment groups (Supplementary Fig. S7B).

## Discussion

Current treatments for prostate cancer, such as ADT, ARPi, and chemotherapy, provide clinical benefits but have notable limitations (e.g., lack of durable response, treatment-related toxicities, development of treatment resistance) that restrict their utility across various disease stages of prostate cancer. These limitations highlight the need for new approaches and novel targets, including hormone-sparing treatments and non-AR targeted therapies, to better address the varied clinical needs of patients with prostate cancer, particularly those with advanced prostate cancer or mCRPC.

Currently, PSMA is the only cell surface protein associated with clinical benefit when therapeutically targeted with an FDA-approved drug [PLUVICTO (lutetium Lu 177 vipivotide tetraxetan)] for advanced prostate cancer. However, PSMA expression is heterogeneous and found in normal tissues such as salivary and lacrimal glands (10). Furthermore, PSMA is not exclusive to prostate cancer; it is also expressed at various levels in other malignancies such as ovarian cancer, thyroid cancer, and non–small cell lung cancer. Additionally, lower levels of PSMA expression have been observed in advanced stages of prostate cancer (30). Our results using a large number of prostate cancer specimens are consistent with the notion of KLK2 as a therapeutic target in prostate cancer, with high levels of KLK2 expression maintained across the prostate disease spectrum from LPC to mCRPC. mCRPC rapid autopsy tissue samples also demonstrated a high prevalence of KLK2 expression despite being heavily pretreated, especially with AR-targeting agents, and close to 90% of those tissue samples had strong IHC staining for KLK2. We also observed higher levels of KLK2 expression compared with PSMA across all disease subsets, including LPC, mHSPC, and mCRPC. In mCRPC samples, a significant proportion of metastatic tumor tissue sites coexpressed KLK2 and PSMA (54% in bone and 61% in lymph nodes). However, a much more significant proportion of lesions expressed KLK2 only compared with PSMA only (bone, 24.1% vs. 9.64%; lymph node, 25% vs. 2.78%, respectively). Note that the positivity rate of PSMA in this study might differ from clinical reports, such as PSMA PET tracer studies, as the definition for target positivity was based on mIF analysis (≥50% positive cells) instead of quantified tracer uptake by maximum standardized uptake value (SUV_max_) or mean standardized uptake value (SUV_mean_).

We also observed that KLK2 expression persisted in mCRPC tissues previously treated with ARPis, potentially attributed to upregulated KLK2 levels by reactivated AR signaling, as previously reported (20). In contrast, increased AR signaling has been shown to downregulate PSMA, suggesting the need for alternative treatments in which AR signaling persists despite reduced serum androgen levels (26). Our results showed that KLK2 expression is largely homogeneous across disease stages in prostate cancer, including advanced prostate cancer, and as such, KLK2 presents unique opportunities for the development of novel therapies in all stages of prostate cancer.

Despite the documented association between KLK2 and prostate cancer progression, as well as its highly prostate-specific expression, KLK2 had previously been thought of as a secreted protease and therefore considered unsuitable for drug targeting. By contrast, the data presented here show that KLK2 is expressed on the surface of prostate cancer cells and can be targeted via multiple therapeutic modalities. Additionally, in a first-in-human phase 0 study, we previously reported that the indium-111 (^111^In)–labeled anti-KLK2 monoclonal antibody, [^111^In]-DOTA-h11B6, demonstrated tumor localization by selectively targeting mCRPC lesions with nominal uptake in healthy organs (31). This is consistent with previously reported preclinical prostate cancer studies of KLK2 distribution (14, 20), highlighting KLK2 as potentially low-risk for on-target, off-tumor toxicity because of its high specificity to prostate and prostate-derived tumors, with minimal-to-no expression in other normal tissues. The preclinical data with ^225^Ac-KLK2 presented here further confirm the therapeutic potential of targeting KLK2 with a ^225^Ac-based radioconjugate for treating prostate cancer.

To further validate KLK2 as a novel target for prostate cancer, we developed two T cell–based therapeutic agents: KLK2 × CD3, a bispecific antibody redirecting CD3^+^ T cells to KLK2-expressing prostate cancer cells, and KLK2-targeted CAR T cells. *In vitro* studies showed that KLK2 × CD3 and KLK2-targeted CAR T cells interacted with KLK2^+^ prostate cancer cells and induced T-cell activation and cytotoxicity. In xenograft mouse models, KLK2 × CD3 and KLK2-targeted CAR T cells exhibited antitumor activity accompanied by CD4^+^ and CD8^+^ T-cell tumor infiltration. A common challenge in characterizing KLK2-targeting therapeutics in preclinical settings is the limited availability of cell lines that endogenously express KLK2 on the cell surface, except for the VCaP cell line. Most preclinical data supporting the development of the three KLK2-targeting therapeutics have been derived from the VCaP cell line, which demonstrates low levels of cell surface KLK2 according to flow cytometry analysis. Therefore, the development of additional cell models that express KLK2 on the cell surface, particularly patient-derived cell lines and materials, will be highly valuable for further elucidating the preclinical activities of these innovative therapeutics.

Taken together, our results show that KLK2 is a promising cell surface target for prostate cancer, addressable by a bispecific antibody (KLK2 × CD3), RLT (^225^Ac-KLK2), and KLK2-targeted CAR T cells. Phase I studies assessing the safety and antitumor activity of KLK2 × CD3 (NCT04898634) and ^225^Ac-KLK2 (NCT04644770), both as monotherapy for patients with mCRPC, are currently ongoing (32, 33).

## Supplementary Material

Supplementary Fig. S1 Supplementary Fig. S1. Representative images of KLK2 staining at different stages of PCa (Localized PCa, n=100; mHSPC, n=98; mCRPC, n=45) by immunohistochemistry. mHSPC, metastatic hormone-sensitive prostate cancer; mCRPC, metastatic castration-resistant prostate cancer; PCa, prostate cancer.

Supplementary Fig. S2 Supplementary Fig. S2. Strong association (spearman rank correlation coefficient=0.74, p=1.6e-06) of proportions of KLK2+ tumor cells in pairs of bone samples (each pair was derived from the same patient). N=34 patients with paired bone samples were used in the analysis. Bone group is defined by the sampling site as backbone, femur, humerus, pelvis, and chest. Samples from the same or different bone groups are identified.

Supplementary Fig. S3 Supplementary Fig. S3. (a) Co-expression of KLK2 and PSMA in patient tissue samples from n=57 patients, as evaluated by multiplex immunofluorescence. Note: *When multiple samples of the same tissue from the same patient were collected, cells were pooled, and samples were marked with asterisk (*) on the top. (b) Representative multiplex immunofluorescence images of each category. CK, cytokeratin; PSMA, prostate-specific membrane antigen.

Supplementary Fig. S4 Supplementary Fig. S4. (a) KLK2×CD3 increased interactions between PCa target cells (VCaP cells expressing Nuclight Red, NLR) and T cells from healthy male donors (n=5). n=3. (b) Staining visualization of KLK2×CD3-induced immune synapse formation between PCa target cell and T-cell. Representative mIF images; n=3.

Supplementary Fig. S5 Supplementary Fig. S5. Representative micrographs of CD8 and CD4 immunostaining of VCaP prostate xenografts treated with 15 mg/kg of null×CD3 control (top panels) or KLK2×CD3 (bottom panels); n=5. Scale bars correspond to 1 cm (main image) and 300 μm (inset).

Supplementary Fig. S6 Supplementary Fig. S6. Schematic illustration of the KLK2 CAR T structure. KLK2 CAR has an scFv-based KLK2-targeting moiety with 4-1BB costimulatory and CD3ζ signaling domains.

Supplementary Fig. S7 Supplementary Fig. S7. in vivo characterization of KLK2-targeted CAR T cells. (a) Administration of KLK2-targeted CAR T cells (5 × 106) to mice with established VCaP tumors induces CAR T-cell expansion and IFNγ release in vivo. Tumor volume, n=10; CAR T expansion and cytokine level, n=5. (b) CD8 and CD4 immunohistochemistry for tumor infiltrating T cells in samples treated with KLK2-targeting CAR T cells. UTD, mock untransduced T cells. Error bars correspond to 300 µm. SF, serum free

## Data Availability

The data sharing policy of Janssen Pharmaceutical Companies of Johnson & Johnson is available at https://www.janssen.com/clinical-trials/transparency. As noted on this site, requests for access to the study data can be submitted through the Yale Open Data Access Project site at http://yoda.yale.edu.
