# Supplementary material for: Human Kallikrein 2: A Novel Lineage-Specific Surface Target in Prostate Cancer
Source: Clin Cancer Res. 2025 Jul 8;31(21):4543–56. doi: 10.1158/1078-0432.CCR-25-0950 (PMC12580770; doi:10.1158/1078-0432.CCR-25-0950)

**Supplementary Fig. S1.** Representative images of KLK2 staining at different stages of PCa (Localized PCa, n=100; mHSPC, n=98; mCRPC, n=45) by immunohistochemistry. mHSPC, metastatic hormone-sensitive prostate cancer; mCRPC, metastatic castration-resistant prostate cancer; PCa, prostate cancer.

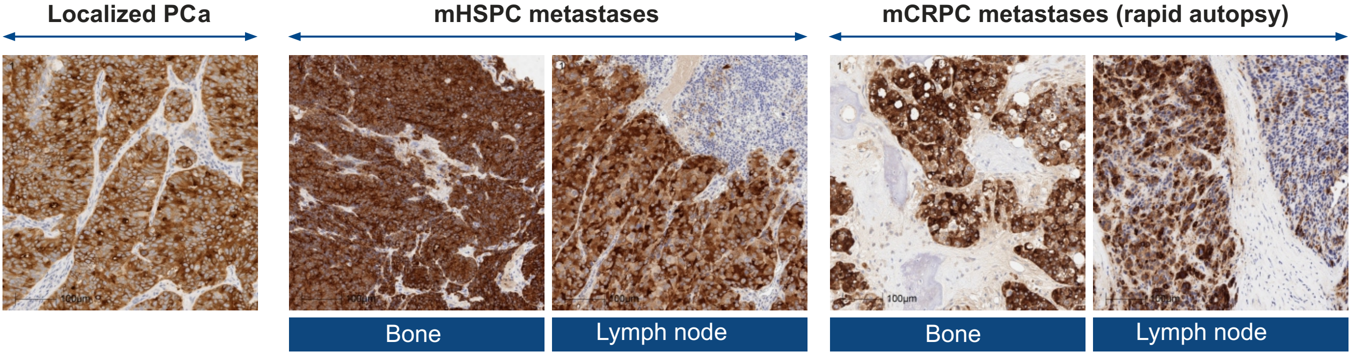

Supplement: Supplementary Fig. S1 — Representative images of KLK2 staining at different stages of PCa (Localized PCa, n=100; mHSPC, n=98; mCRPC, n=45) by immunohistochemistry. mHSPC, metastatic hormone-sensitive prostate cancer; mCRPC, metastatic castration-resistant prostate cancer; PCa, prostate cancer. [file ccr-25-0950_supplementary_fig.s1_suppsf1.pdf]
