# Supplementary material for: Human Kallikrein 2: A Novel Lineage-Specific Surface Target in Prostate Cancer
Source: Clin Cancer Res. 2025 Jul 8;31(21):4543–56. doi: 10.1158/1078-0432.CCR-25-0950 (PMC12580770; doi:10.1158/1078-0432.CCR-25-0950)

Supplementary Fig. S2. Strong association (spearman rank correlation coefficient=0.74, p=1.6e-06) of proportions of KLK2+ tumor cells in pairs of bone samples (each pair was derived from the same patient). N=34 patients with paired bone samples were used in the analysis. Bone group is defined by the sampling site as backbone, femur, humerus, pelvis, and chest. Samples from the same or different bone groups are identified.

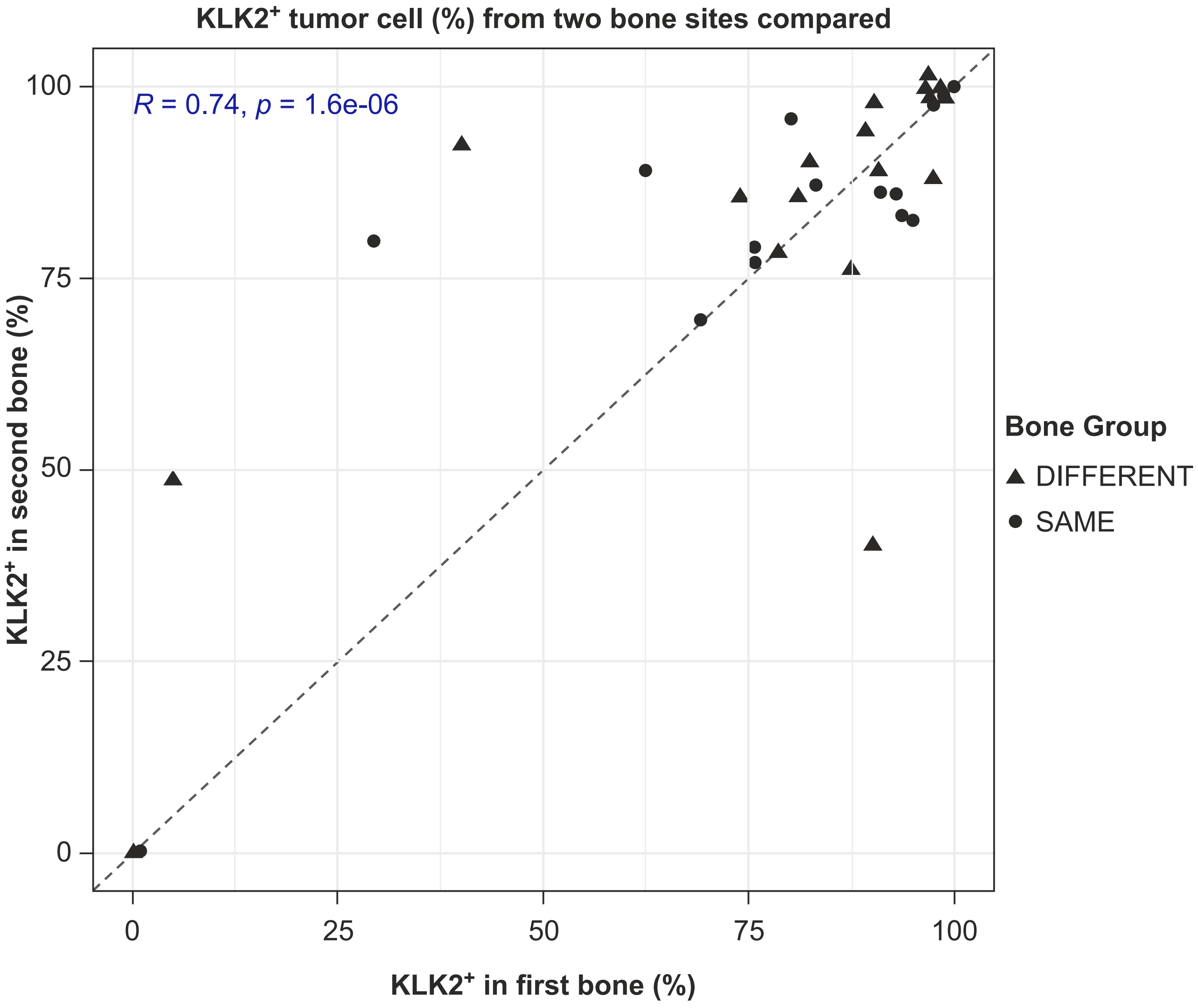

Supplement: Supplementary Fig. S2 — Strong association (spearman rank correlation coefficient=0.74, p=1.6e-06) of proportions of KLK2+ tumor cells in pairs of bone samples (each pair was derived from the same patient). N=34 patients with paired bone samples were used in the analysis. Bone group is defined by the sampling site as backbone, femur, humerus, pelvis, and chest. Samples from the same or different bone groups are identified. [file ccr-25-0950_supplementary_fig.s2_suppsf2.pdf]
