# Supplementary material for: Human Kallikrein 2: A Novel Lineage-Specific Surface Target in Prostate Cancer
Source: Clin Cancer Res. 2025 Jul 8;31(21):4543–56. doi: 10.1158/1078-0432.CCR-25-0950 (PMC12580770; doi:10.1158/1078-0432.CCR-25-0950)

Supplementary Fig. S3. (a) Co-expression of KLK2 and PSMA in patient tissue samples from n=57 patients, as evaluated by multiplex immunofluorescence. Note: *When multiple samples of the same tissue from the same patient were collected, cells were pooled, and samples were marked with asterisk (*) on the top. **(b)** Representative multiplex immunofluorescence images of each category. CK, cytokeratin; PSMA, prostate-specific membrane antigen.

(a)

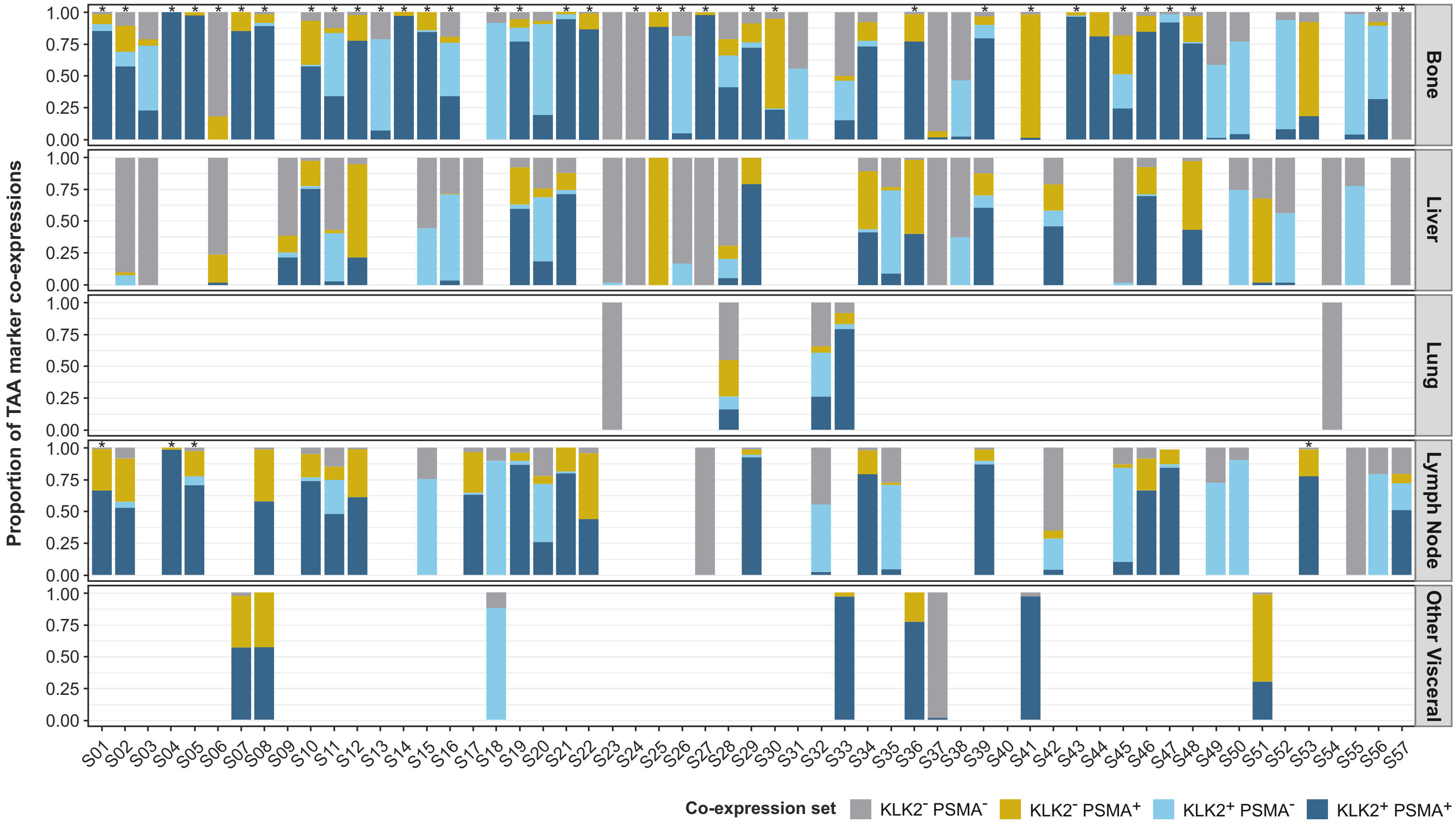

(b)

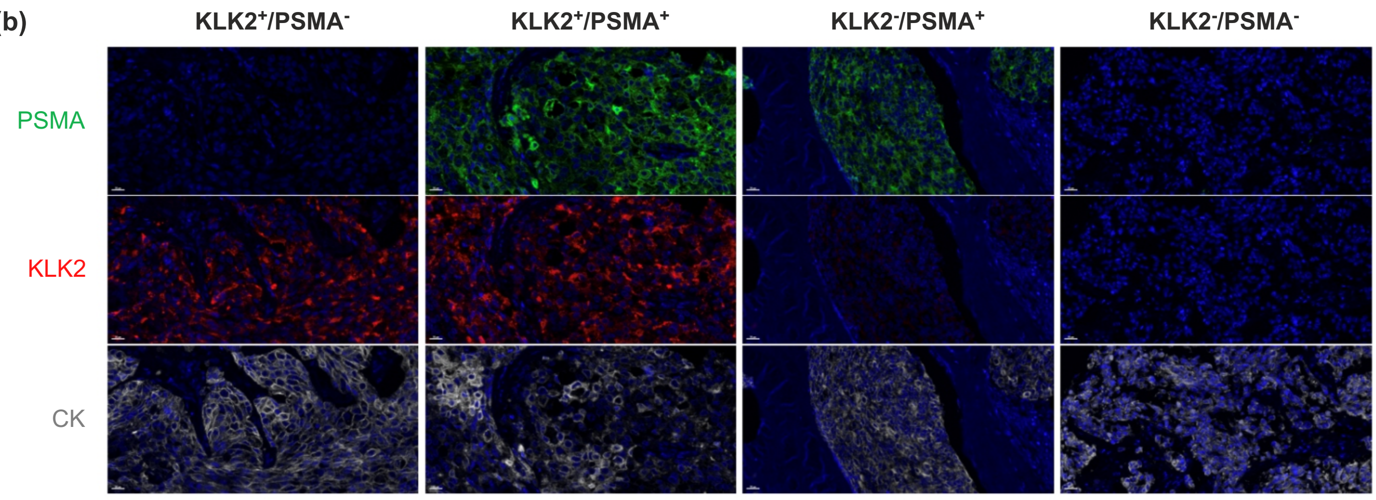

Supplement: Supplementary Fig. S3 — (a) Co-expression of KLK2 and PSMA in patient tissue samples from n=57 patients, as evaluated by multiplex immunofluorescence. Note: *When multiple samples of the same tissue from the same patient were collected, cells were pooled, and samples were marked with asterisk (*) on the top. (b) Representative multiplex immunofluorescence images of each category. CK, cytokeratin; PSMA, prostate-specific membrane antigen. [file ccr-25-0950_supplementary_fig.s3_suppsf3.pdf]
