# Supplementary material for: Human Kallikrein 2: A Novel Lineage-Specific Surface Target in Prostate Cancer
Source: Clin Cancer Res. 2025 Jul 8;31(21):4543–56. doi: 10.1158/1078-0432.CCR-25-0950 (PMC12580770; doi:10.1158/1078-0432.CCR-25-0950)

Supplementary Fig. S4. (a) KLK2×CD3 increased interactions between PCa target cells (VCaP cells expressing Nuclight Red, NLR) and T cells from healthy male donors (n=5). n=3. **(b)** Staining visualization of KLK2×CD3-induced immune synapse formation between PCa target cell and T-cell. Representative mIF images; n=3.

(a)

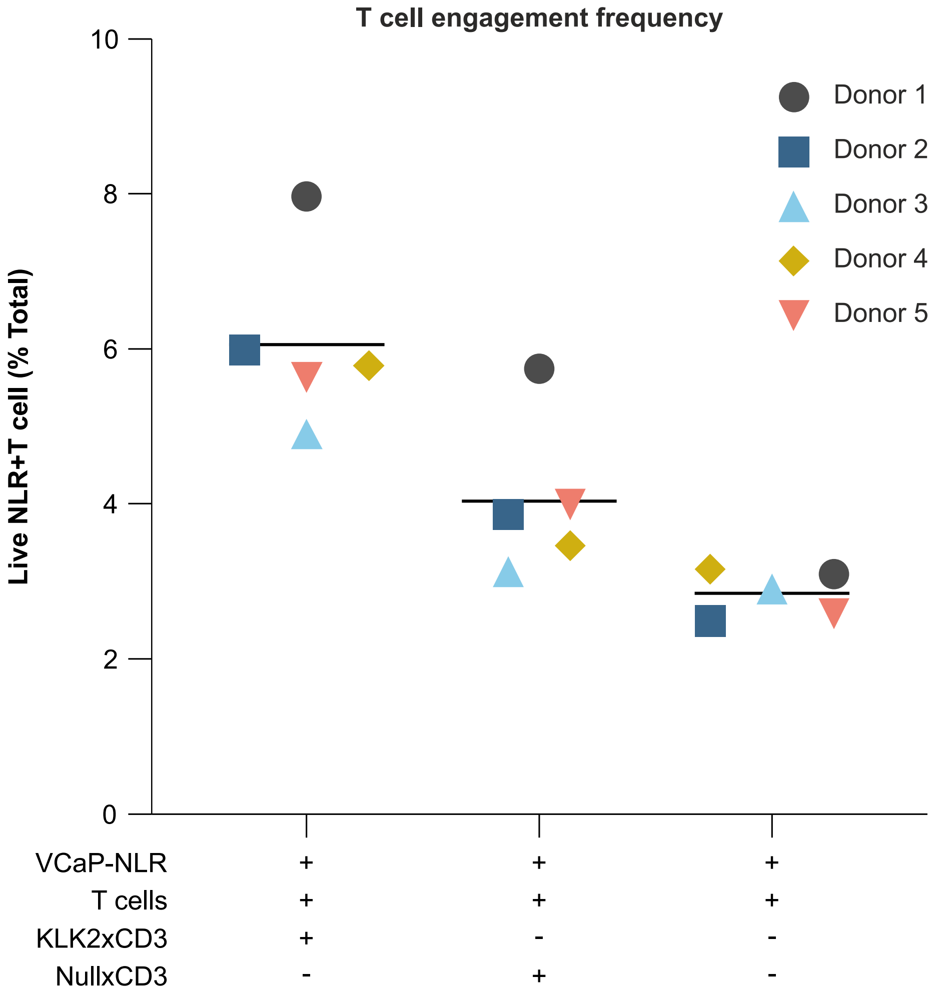

(b)

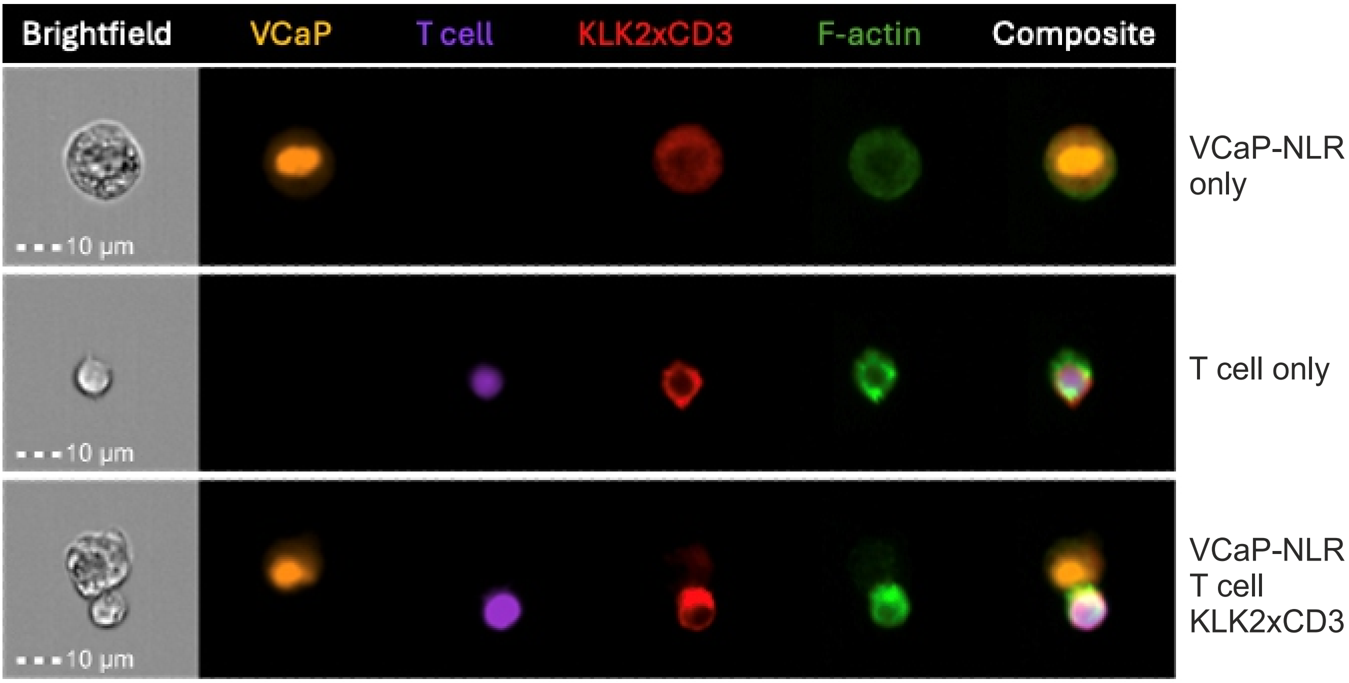

Supplement: Supplementary Fig. S4 — (a) KLK2×CD3 increased interactions between PCa target cells (VCaP cells expressing Nuclight Red, NLR) and T cells from healthy male donors (n=5). n=3. (b) Staining visualization of KLK2×CD3-induced immune synapse formation between PCa target cell and T-cell. Representative mIF images; n=3. [file ccr-25-0950_supplementary_fig.s4_suppsf4.pdf]
