# Supplementary material for: Human Kallikrein 2: A Novel Lineage-Specific Surface Target in Prostate Cancer
Source: Clin Cancer Res. 2025 Jul 8;31(21):4543–56. doi: 10.1158/1078-0432.CCR-25-0950 (PMC12580770; doi:10.1158/1078-0432.CCR-25-0950)

Supplementary Fig. S5. Representative micrographs of CD8 and CD4 immunostaining of VCaP prostate xenografts treated with 15 mg/kg of null×CD3 control (top panels) or KLK2×CD3 (bottom panels); n=5. Scale bars correspond to 1 cm (main image) and 300 μm (inset).

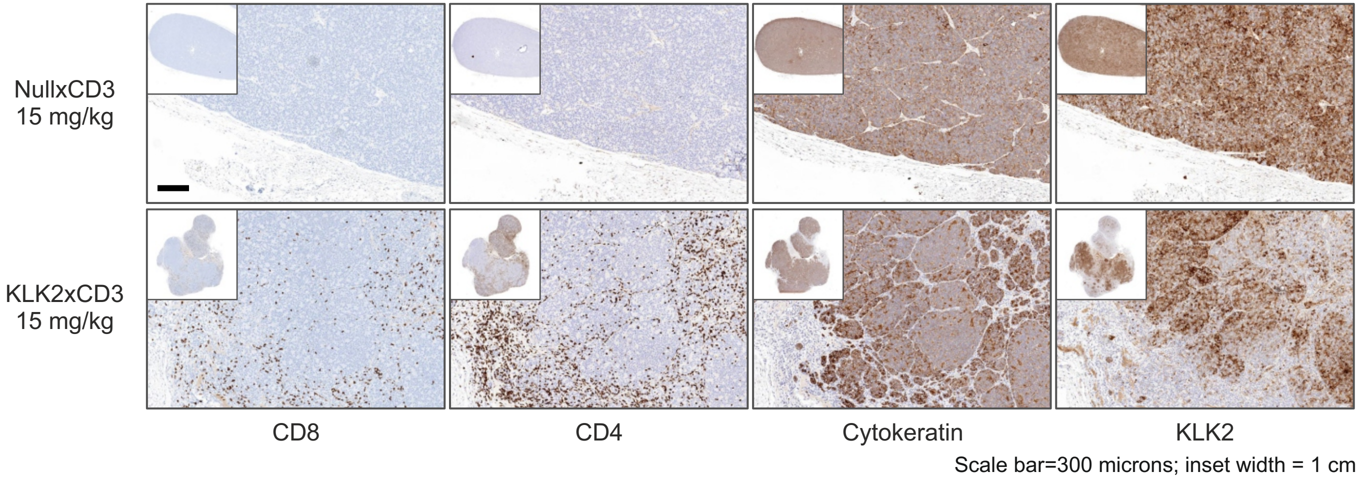

Supplement: Supplementary Fig. S5 — Representative micrographs of CD8 and CD4 immunostaining of VCaP prostate xenografts treated with 15 mg/kg of null×CD3 control (top panels) or KLK2×CD3 (bottom panels); n=5. Scale bars correspond to 1 cm (main image) and 300 μm (inset). [file ccr-25-0950_supplementary_fig.s5_suppsf5.pdf]
