# Supplementary material for: Human Kallikrein 2: A Novel Lineage-Specific Surface Target in Prostate Cancer
Source: Clin Cancer Res. 2025 Jul 8;31(21):4543–56. doi: 10.1158/1078-0432.CCR-25-0950 (PMC12580770; doi:10.1158/1078-0432.CCR-25-0950)

Supplementary Fig. S6. Schematic illustration of the KLK2 CAR T structure. KLK2 CAR has an scFv-based KLK2-targeting moiety with 4-1BB costimulatory and CD3ζ signaling domains.

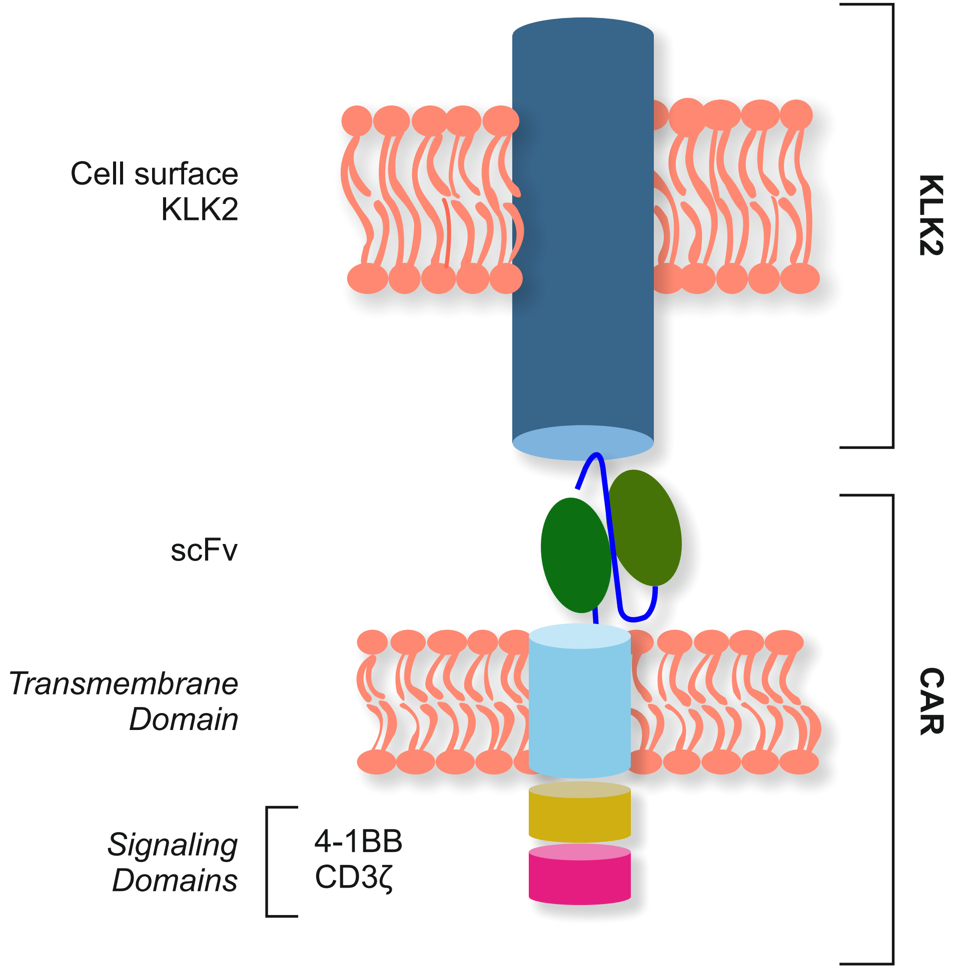

Supplement: Supplementary Fig. S6 — Schematic illustration of the KLK2 CAR T structure. KLK2 CAR has an scFv-based KLK2-targeting moiety with 4-1BB costimulatory and CD3ζ signaling domains. [file ccr-25-0950_supplementary_fig.s6_suppsf6.pdf]
