# Supplementary material for: Human Kallikrein 2: A Novel Lineage-Specific Surface Target in Prostate Cancer
Source: Clin Cancer Res. 2025 Jul 8;31(21):4543–56. doi: 10.1158/1078-0432.CCR-25-0950 (PMC12580770; doi:10.1158/1078-0432.CCR-25-0950)

Supplementary Fig. S7. in vivo characterization of KLK2-targeted CAR T cells. (a) Administration of KLK2-targeted CAR T cells (5x106) to mice with established VCaP tumors induces CAR T-cell expansion and IFNγ release in vivo. Tumor volume, n=10; CAR T expansion and cytokine level, n=5. **(b)** CD8 and CD4 immunohistochemistry for tumor infiltrating T cells in samples treated with KLK2-targeting CAR T cells. UTD, mock untransduced T cells. Error bars correspond to 300 µm. SF, serum free.

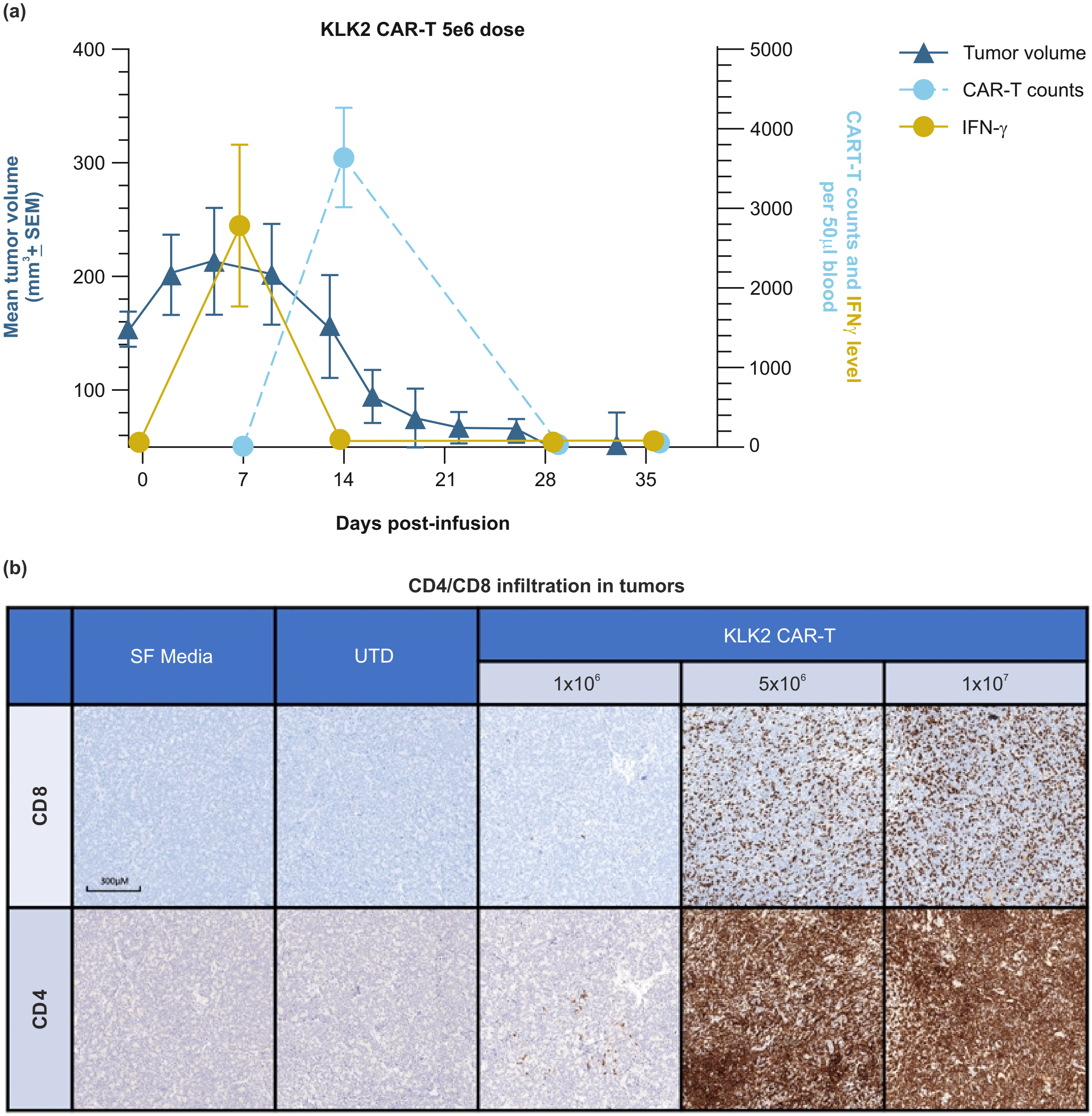

Supplement: Supplementary Fig. S7 — in vivo characterization of KLK2-targeted CAR T cells. (a) Administration of KLK2-targeted CAR T cells (5 × 10^6^) to mice with established VCaP tumors induces CAR T-cell expansion and IFNγ release in vivo. Tumor volume, n=10; CAR T expansion and cytokine level, n=5. (b) CD8 and CD4 immunohistochemistry for tumor infiltrating T cells in samples treated with KLK2-targeting CAR T cells. UTD, mock untransduced T cells. Error bars correspond to 300 µm. SF, serum free [file ccr-25-0950_supplementary_fig.s7_suppsf7.pdf]
